# Risk factors associated with tumor upstaging in melanomas treated with Mohs micrographic surgery with melanocytic immunohistochemistry

**DOI:** 10.1016/j.jdin.2024.02.006

**Published:** 2024-03-26

**Authors:** Thomas Trischman, Anagha Bangalore Kumar, Eucabeth M. Asamoah, Austin Todd, Nahid Y. Vidal, Addison M. Demer

**Affiliations:** a Department of Dermatology, Mayo Clinic, Rochester, Minnesota; b Department of Quantitative Health Sciences, Mayo Clinic, Rochester, Minnesota; c Division of Dermatologic Surgery, Department of Dermatology, Mayo Clinic, Rochester, Minnesota

**Keywords:** dermatologic surgery, lentigo maligna, melanoma, melanoma in situ, melanoma upstaging, melanoma upstaging with Mohs, Mohs for melanoma, Mohs micrographic surgery, Mohs with immunohistochemistry, upstaging, wide local excision

## Abstract

**Background:**

Mohs micrographic surgery with melanocytic immunohistochemistry (MMS-I) is increasingly utilized for special site melanoma treatment. Yet, frequency and risk factors associated with upstaging of all-stage cutaneous melanomas treated with MMS-I remain undefined.

**Objective:**

Determine upstaging frequency and factors associated with tumor upstaging for all-stage melanomas treated with MMS-I.

**Methods:**

In this retrospective, single-center case series, all cases of invasive and in situ melanoma treated with MMS-I between 2008 and 2018 were reviewed. Patient and tumor characteristics were recorded and compared between tumors that were and were not upstaged from their initial T stage.

**Results:**

Of the 962 melanoma MMS-I cases identified, 44 (4.6%) were upstaged, including 5.6% of in situ and 2.5% of invasive tumors. Risk factors for upstaging included lack of excisional intent at the time of initial biopsy (*P* < .01), nonlentigo maligna subtype (*P* = .03), female sex (*P* = .02), and initial in situ diagnosis (*P* = .03). Nonstatistically significant characteristics evaluated included patient age (*P* = .97), initial Breslow depth (*P* = .18), and biopsy type (*P* = .24).

**Limitations:**

Retrospective study design.

**Conclusions:**

All-stage cutaneous melanomas treated with MMS-I are associated with low upstaging rates. Tumor upstaging is associated with lack of excisional intent, female sex, and in situ tumors.

**Capsule Summary**

• While uncommon (<5% of tumors), melanoma upstaging at time of Mohs surgery has important implications for treatment and prognosis.
• To ensure appropriate management and counseling, clinicians should be aware of risk factors for melanoma upstaging including female sex, initial in situ diagnosis, and lack of excisional intent on initial biopsy.

## Introduction

Mohs micrographic surgery (MMS), and particularly, Mohs micrographic surgery with melanocytic immunohistochemistry (MMS-I) is frequently being utilized to treat both in situ and invasive melanomas on special sites.^1^ There is a growing body of data supporting MMS-I as an excellent treatment modality for both in situ and invasive melanomas, with low rates of local recurrence, nodal recurrence, distant recurrence, metastasis, and death.^2^, ^3^, ^4^, ^5^, ^6^, ^7^, ^8^, ^9^, ^10^, ^11^ MMS-I allows for comprehensive margin assessment prior to immediate surgical defect repair. Appropriate tumor staging prior to MMS-I is essential to ensure arrangement for sentinel lymph node biopsy and additional staging workup if indicated.^12^

Tumor stage is primarily determined by Breslow thickness, which in turn depends heavily on biopsy technique. According to the most recent clinical practice guidelines from the American Academy of Dermatology, the preferred biopsy technique for a suspected melanoma is a narrow (1–3 mm margin) excisional/complete biopsy encompassing the entire breadth of the lesion and of sufficient depth to avoid transection at the base. This can be accomplished with fusiform/elliptical, punch, or shave/saucerization techniques. These same guidelines state that incomplete or partial sampling is acceptable in certain situations, such as when lesions are on the face, very large, or of diagnostic uncertainty. Furthermore, an additional narrow margin excisional biopsy may be considered after initial partial biopsy if the initial biopsy was inadequate for diagnosis or microstaging unless the initial specimen met criteria for sentinel lymph node biopsy.^3^ Lesions described by these guidelines represent a large proportion of melanomas treated with MMS-I.

Previous studies evaluating special site and/or MMS-treated melanomas have reported a range from 4% to 21.7% of tumor upstaging.^13^, ^14^, ^15^, ^16^, ^17^ Frequency and risk factors associated with upstaging of all-stage cutaneous melanomas treated with MMS-I remain undefined. We sought to evaluate frequency of upstaging in all-stage melanomas treated with MMS-I and risk factors associated with upstaging including biopsy type.

## Methods

In this institutional review board–exempt, single-center, retrospective case series, we identified all cases of melanoma in situ and all-stage invasive melanoma in adults treated with MMS-I between 2008 and 2018. Data concerning demographics, initial biopsy information, characteristics of the melanoma diagnosed, and tumor upstaging status were collected. Observed and expected frequencies of upstaged cases were compared with those of nonupstaged cases using Pearson’s chi-squared test for categorical variables, and the Wilcoxon rank sum test was used for continuous variables.

## Results

Nine hundred sixty-two cases of melanoma in 926 unique patients were identified for inclusion. Among these patients, 663 were male (71.7%) who had a total of 692 tumors (71.9%); 263 were female (28.3%) who had a total of 270 tumors (28.1%). Six hundred thirty-nine tumors were diagnosed as in situ melanoma on the initial biopsy (66.4%), while 323 were originally diagnosed as invasive melanoma (33.6%). Immunostaining with Melanoma Antigen Recognized by T cells or Melan-A or microphthalmia-associated transcription factor was used for all cases, with Melanoma Antigen Recognized by T cells being most commonly used. Most tumors (87.7%) in this study were lentigo maligna (LM) subtype of melanoma, followed by superficial spreading subtype at 8.4%. The average Breslow thickness for invasive tumors at the time of initial diagnosis was 0.5 ± 0.3 mm. By initial T-stage, 639 melanomas were in situ (66.4%), 293 were T1a (30.5%), 15 were T1b (1.6%), 10 were T2a (1.0%), 2 were T2b (0.2%), and 3 were T3a (0.3%). Forty-four tumors (4.6%) were upstaged, among which 36 were originally in situ tumors upstaged to invasive tumors (5.6% upstaging rate for tumors initially diagnosed as in situ), and 8 of which were invasive tumors upstaged to a higher T stage (2.5% upstaging rate for invasive tumors). Among the 44 upstaged tumors, 39 (88.6%) were upstaged at the time of MMS-I, 37 (94.9%) of which were upstaged from frozen and/or permanent H&E sectioning of the Mohs debulk specimen. The remaining 5 upstaged tumors were upstaged prior to MMS-I after multiple biopsies were performed. Among upstaged melanomas, 8 (18.2%) were upstaged to T1b or higher. Among those 8, additional workup or changes to management were considered in 7 (87.5%), but due to age, comorbidities, or patient preference, an actual change in management or workup only took place in 3 (37.5%).

Results and analyses are summarized with patient demographics and clinical characteristics in Table I, and tumor characteristics in Table II. Statistically significant differences in expected versus observed proportions of upstaged melanomas were found when comparing patient sex (more females had upstaged tumors than expected), tumors initially diagnosed as in situ versus invasive melanomas (more melanomas initially diagnosed as in situ were upstaged than expected), melanomas that were partially biopsied compared with melanomas where the entire pigmented lesion was attempted to be sampled (more melanomas that were partially biopsied were upstaged than expected), and various tumor subtypes (more melanomas that were not LM subtype were upstaged than expected). For melanomas that were invasive at the time of the initial diagnosis, higher Breslow thickness was associated with upstaging. There were no statistically significant findings when we evaluated the relationship between upstaging and age at diagnosis, anatomic site, primary versus recurrent melanomas, biopsy method, initial Breslow depth, tumor transection on the initial pathology report, or tumor extension to peripheral biopsy margins on the initial pathology report.

**Table I:** Patient demographics, melanoma clinical and pathologic characteristics, and associated results from analyses of these variables

	Upstaged (4.6%)	Not upstaged (95.4%)	Total	*P*-value
Patient demographics				
Patients	43 (4.6%)	883 (95.4%)	926	
Tumors	44 (4.6%)	918 (95.4%)	962	
Sex (males:females)	25 (56.8%):19 (43.8%)	638 (72.3%):244 (27.7%)	663 (71.6%):263 (28.4%)	.02∗
Age (mean ± SD, median)	70 ± 11, 71	69 ± 12, 70	69 ± 12, 70	.97†
Clinical characteristics				
Location				.90∗
Head and neck	41 (93.2%)	851 (92.7%)	892 (92.7%)	
Upper extremity (not hand)	1 (2.3%)	16 (1.7%)	17 (1.8%)	
Lower extremity (not foot)	0 (0%)	14 (1.5%)	14 (1.5%)	
Chest and abdomen	0 (0%)	7 (0.8%)	7 (0.7%)	
Back	0 (0%)	9 (1.0%)	9 (0.9%)	
Hands and feet (not nail unit)	2 (4.5%)	19 (2.1%)	21 (2.2%)	
Nail unit	0 (0%)	1 (0.1%)	1 (0.1%)	
Genitalia	0 (0%)	1 (0.1%)	1 (0.1%)	
Primary or recurrent				.55∗
Primary tumors	41 (93.2%)	882 (96.1%)	923 (96.0%)	
Recurrent tumors	3 (6.8%)	34 (3.7%)	37 (3.8%)	
Unable to determine	0 (0%)	2 (0.2%)	2 (0.2%)	
Entire visible lesion biopsied (excisional intent)				<.01∗
Yes	8 (18.2%)	470 (51.2%)	478 (49.7%)	
No	26 (59.2%)	389 (42.4%)	415 (43.1%)	
Unknown	10 (22.7%)	59 (6.4%)	69 (7.2%)	
Biopsy type				.24∗
Shave	28 (63.6%)	579 (63.1%)	607 (63.1%)	
Punch	11 (25.0%)	143 (15.6%)	154 (16.0%)	
Unknown	5 (11.4%)	139 (15.1%)	144 (15.0%)	
Excision	0 (0%)	56 (6.1%)	56 (5.8%)	
Incisional	0 (0%)	1 (0.1%)	1 (0.1%)	

∗ Calculated using Pearson's chi-square test.

† Calculated using Wilcoxon rank sum test.

**Table II:** Melanoma pathology and perioperative characteristics and associated results of analyses

	Upstaged (4.6%)	Not upstaged (95.4%)	Total	*P*-value
Mean initial Breslow depth (invasive only)	0.8 ± 0.5 (*n* = 9)	0.4 ± 0.3 (*n* = 315)	0.5 ± 0.3 (*n* = 324)	<.01∗
In situ or invasive on initial biopsy				<.01†
Invasive	8 (18.2%)	315 (34.3%)	323 (33.6%)	
In situ	36 (81.8%)	603 (65.7%)	639 (66.4%)	
Tumor subtype				<.01†
Superficial spreading	2 (4.5%)	79 (8.6%)	81 (8.4%)	
Lentigo maligna	34 (77.3%)	810 (88.2%)	844 (87.7%)	
Nodular	2 (4.5%)	1 (0.1%)	3 (0.3%)	
Acral lentiginous	2 (4.5%)	17 (1.9%)	19 (2.0%)	
Other/unspecified	4 (9.1%)	11 (1.2%)	15 (1.6%)	
T stage (initial biopsy)‡				
Tis	36 (81.8%)	603 (65.7%)	639 (66.4%)	
T1a	5 (11.4%)	288 (31.4%)	293 (30.5%)	
T1b	1 (2.3%)	14 (1.5%)	15 (1.6%)	
T2a	2 (4.5%)	8 (0.9%)	10 (1.0%)	
T2b	0 (0%)	2 (0.2%)	2 (0.2%)	
T3a	0 (0%)	3 (0.3%)	3 (0.3%)	
T stage (final)				
Tis	0 (0%)	603 (65.7%)	603 (62.7%)	
T1a	36 (81.8%)	290 (31.6%)	326 (33.9%)	
T1b	2 (4.5%)	13 (1.4%)	15 (1.6%)	
T2a	1 (2.3%)	7 (0.8%)	8 (0.8%)	
T2b	0 (0%)	2 (0.2%)	2 (0.2%)	
T3a	3 (6.8%)	3 (0.3%)	6 (0.6%)	
T4a	2 (4.5%)	0 (0%)	2 (0.2%)	
Transected deep margin				.15†
Yes	5 (11.4%)	162 (17.6%)	167 (17.4%)	
No	38 (86.4%)	752 (81.9%)	790 (82.1%)	
Unspecified	1 (2.3%)	4 (0.4%)	5 (0.5%)	
Extension to peripheral margin				.34†
Yes	40 (90.1%)	768 (83.7%)	808 (84.0%)	
No	2 (4.5%)	107 (11.7%)	109 (11.3%)	
Unspecified	2 (4.5%)	43 (4.7)	45 (4.7%)	
Tumor debulked and sent for permanent histopathology processing				<.01†
Yes	43 (97.7%)	603 (65.7%)	646 (67.2%)	
No	1 (2.3%)	315 (34.3%)	316 (32.8%)	

∗ Calculated using Wilcoxon rank sum test.

† Calculated using Pearson's chi-square test.

‡ All T stages in this publication use the eighth edition of the American Joint Committee on Cancer staging for cutaneous melanoma.

## Discussion

Multiple large studies have been published describing risk factors associated with upstaging of melanomas treated with excision, while relatively fewer have been published for melanomas treated with MMS-I. Upstaging rates in prior studies have varied widely. The overall rate of upstaging in Etzkorn and colleagues’ study of 1332 in situ and all-stage invasive melanomas treated with wide local excision (WLE) was 3.9%, but when they examined the upstaging rate for both the head and neck and hands or feet, this rate was found to be much higher at 12%.^13^ In a retrospective series by Baum and colleagues describing 624 in situ melanomas on the head and neck, some of which were treated with conventional WLE and some of which were treated with MMS-I, the overall upstaging rate was 4%.^17^ In their comprehensive review of publications concerning complications of in situ and invasive melanomas on all sites treated with WLE, Miller and colleagues coined a “rule of 10s” to describe the average 10% rate of upstaging, 10% risk of positive margins, 10% risk of local recurrence, and 10-fold risk of requiring a flap or graft repair in melanomas treated with WLE on special sites. They contrasted this to the “rule of 2s” for melanomas on the trunk and proximal extremities, with the same complications described above but associated with much lower rates of 2% or two-fold, respectively.^18^

There have been some previous studies evaluating upstaging frequency and risk factors in melanomas treated with MMS-I. In their study of 117 in situ melanomas treated with MMS, 10 (8.5%) of which were upstaged, Levoska and colleagues described how upstaging was unlikely to require a change in management of melanomas that were treated with MMS-I, but this study did not evaluate risk factors associated with upstaging in tumors treated with MMS-I.^19^ Iorizzo 3rd and colleagues described 14 upstaged melanomas out of 173 (8.1%) total tumors treated with MMS-I, but found no differences in studied characteristics between upstaged and nonupstaged melanomas.^20^

Our study describes the frequency and associated risk factors related to upstaging for in situ and all-stage invasive melanomas treated with MMS-I. Notably, we identified a positive association between tumor upstaging and lack of excisional intention on initial biopsy, non-LM melanoma histologic subtypes, female gender, and initial in situ diagnosis. We also found an overall upstaging rate of 4.6% for melanomas treated with MMS-I, with an upstaging rate of 5.6% for melanomas initially diagnosed as in situ, and 2.5% for melanomas initially diagnosed as invasive.

The finding that biopsy technique is less important than biopsy intent is not surprising and in line with one of the most basic principles of performing skin biopsies for cutaneous neoplasms: partial biopsies may not be representative of the entire lesion. This principle is reflected in national treatment guidelines regarding recommended biopsy techniques for suspected melanomas, which suggest a narrow-margin excisional biopsy of sufficient depth using a fusiform/elliptical or punch excision or deep shave/saucerization removal to depth below the anticipated plane of the lesion, with exceptions existing describing when a partial sample or an additional biopsy after the initial biopsy may be considered. In this study, 415 (43.1%) of melanomas were partially sampled at the time of initial biopsy. It is unclear if this was due to sensitive anatomic locations, lesion breadth, suboptimal biopsy technique, or a combination of factors. The finding that sending a central debulk for permanent sectioning is associated with upstaging is also intuitive and reinforces existing guidelines from the American Academy of Dermatology and the research the guidelines are based upon.^12^^,^^21^ The upstaging rate we found in our study (4.6%) in melanomas treated with MMS-I is overall favorable and is comparable to upstaging rates (ranging from 4% to 8.5%) from prior studies evaluating melanomas on the head and neck treated with MMS-I.^17^^,^^19^^,^^20^

Interestingly, the largest study of risk factors related to melanoma upstaging with WLE found that male sex was associated with increased risk of upstaging, whereas our study showed the opposite.^22^ Perhaps this is due to increased concern for cosmesis in females when suspicious lesions on the head and neck are biopsied, with a resultant increased likelihood of obtaining a nonrepresentative partial sample. The aforementioned study also identified an association between head/neck tumor location and tumor upstaging, whereas our study did not. Of note, the vast majority of tumors treated in our cohort were located on head/neck sites, thus limiting value of anatomic site comparison between subgroups. There were likely an insufficient number of nonhead/neck tumors present in this study to identify a statistically significant difference if one exists. In contrast, both studies were concordant in showing an increased risk of upstaging with nodular and acral lentiginous subtypes.

Limitations of our study are its retrospective nature, single-center cohort, lack of comparative WLE group, and absence of accurate patient race, ethnicity, and socioeconomic data which may have provided insight into potential disparities that could be addressed to improve outcomes.

## Conclusions

Special site melanomas are increasingly treated with MMS-I given comprehensive and reliable same-day margin assessment, healthy tissue-sparing nature when compared to standard wide local excision, high rates of patient satisfaction, and excellent reported local recurrence, metastasis, and survival outcomes. Melanomas treated with MMS-I are occasionally upstaged between the time of initial biopsy and the end of the treatment process, which has the potential to drastically change prognosis and management, though in this study upstaging only changed management in 3 cases. Ideally, melanomas should be properly staged prior to treatment to ensure the most appropriate treatment is being pursued and that additional workup is performed with appropriate timing if indicated. We identified a low overall upstaging rate of 4.6% for in situ and all-stage invasive melanomas treated with MMS-I. Risk factors associated with upstaging included female sex, non-LM tumor subtype, initial in situ diagnosis, and melanomas biopsied without excisional intent. These risk factors should be considered when counseling patients on risk of intra/postoperative upstaging. Furthermore, the results affirm national treatment guidelines which emphasize narrow-margin excisional biopsy of melanocytic lesions at the time of initial biopsy and emphasize the importance of perioperative debulk prior to Mohs treatment for cutaneous melanoma.

## Conflicts of interest

None disclosed.
